# Longitudinal DNA methylation in parent–infant pairs impacted by intergenerational social adversity: An RCT of the Michigan Model of Infant Mental Health Home Visiting

**DOI:** 10.1002/brb3.70035

**Published:** 2024-09-18

**Authors:** Rebekah L. Petroff, Jennifer Jester, Jessica Riggs, Emily Alfafara, Katherine Springer, Natalie Kerr, Meriam Issa, Alanah Hall, Katherine Rosenblum, Jaclyn M. Goodrich, Maria Muzik, the Michigan Collaborative for Infant Mental Health Research

**Affiliations:** ^1^ Department of Environmental Health Sciences, School of Public Health University of Michigan Ann Arbor Michigan USA; ^2^ Department of Psychiatry Michigan Medicine Ann Arbor Michigan USA; ^3^ Department of Obstetrics & Gynecology Michigan Medicine Ann Arbor Michigan USA; ^4^ Department of Pediatrics Michigan Medicine Ann Arbor Michigan USA

**Keywords:** DNA methylation, early life adversity, epigenetics, Infant Mental Health, parent–child psychotherapy, RCT trial

## Abstract

**Introduction:**

Early childhood development is a strong predictor of long‐term health outcomes, potentially mediated via epigenetics (DNA methylation). The aim of the current study was to examine how childhood experiences, punitive parenting, and an intergenerational psychotherapeutic intervention may impact DNA methylation in young children and their mothers.

**Methods:**

Mothers and their infants/toddlers between 0 and 24 months were recruited at baseline (*n* = 146, 73 pairs) to participate in a randomized control trial evaluating the effectiveness of The Michigan Model of Infant Mental Health Home Visiting (IMH‐HV) parent–infant psychotherapy compared to treatment as usual. Baseline and 12‐month post‐enrollment data were collected in the family's home and included self‐report questionnaires, biological saliva samples, home environment observation, video‐taped parent–child interaction, and audio‐recorded interviews. Saliva DNA methylation was measured at the genes, nuclear receptor subfamily 3 group C member 1 (*NR3C1*), solute carrier family 6 member 4 (*SLC6A4*), brain‐derived neurotrophic factor (*BDNF*), and the genetic element, long interspersed nuclear element‐1 (LINE1).

**Results:**

For mothers, baseline methylation of *BDNF*, *SLC6A4*, *NR3C1*, or LINE1 was largely not associated with baseline measures of their childhood adversity, adverse life experiences, demographic characteristics related to structurally driven inequities, or to IMH‐HV treatment effect. In infants, there were suggestions that methylation in *SLC6A4* and LINE1 was associated with parenting attitudes. Infant *BDNF* methylation suggested an overall decrease in response to IMH‐HV psychotherapy over 12 months.

**Conclusions:**

Overall, our findings suggest that the epigenome in infants and young children may be sensitive to both early life experiences and parent–infant psychotherapy.

## Graphical abstract

Families at greater risk for disruptions to the parent–child relationship received either weekly Infant Mental Health Home Visiting parent–infant psychotherapy or treatment as usual for 12 months. DNA methylation, which controls gene expression, was measured at stress responsive genes. Children's saliva DNA methylation at *SLC6A4* and LINE1 was associated with parenting attitudes, and *BDNF* methylation also decreased in response to therapy.

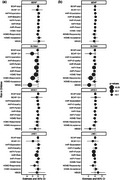

## 1 INTRODUCTION

Early life experiences and adversity in childhood and adulthood can impact health and disease throughout life. In adults, childhood adversity is associated with an increased risk of substance use, poor mental health, chronic diseases, and premature mortality (Hughes et al., 2017). Children of parents with childhood adversity are also at increased risk for developmental delays (Folger et al., 2018), suggesting that there is an intergenerational link between parental childhood adversity and negative health outcomes in both parent and child. These factors are also impacted by broad structural factors and are collectively called social determinants of health (Braveman & Gottlieb, 2014; Braveman et al., 2011; Denburg & Daneman, 2010).

This intergenerational relationship is proposed to be driven by differences in the structural (e.g., policies, racism, and city planning), social (e.g., parenting and social norms), and biological environment (e.g., the genome), or some combination of the above (Kellermann, 2001). A potential intermediate or mediator of these factors is the epigenome, the collection of chemical marks and small molecules that is responsive to the environment and controls gene expression without changing the DNA sequence. Differences in the social environment can lead to alterations of these epigenetic marks; subsequent expression changes of various biological system genes, which are responsible for maintaining physiological stability, may be responsible for mental health or disease risk throughout development and into adulthood (Barker, 2007). The most studied epigenetic mark, DNA methylation, has been associated with differences in vulnerability to psychiatric diseases later in life (Klengel & Binder, 2015). Other studies have retrospectively found that some types of childhood adversity are associated with DNA methylation in adulthood (Neves et al., 2021), and DNA methylation in adults may modify response to psychotherapy (Penadés et al., 2020). In children, measures of early life adversity have also been related to differences in DNA methylation (Non et al., 2016; Weder et al., 2014).

Our knowledge of the relationship between the social environment and the epigenome is largely based on results from retrospective cohort or cross‐sectional studies in adults (Cerutti et al., 2021). However, because the human body is rapidly growing and developing (Wikenius et al., 2019), infancy and toddlerhood (and the developing epigenome at these ages) are unique compared to other developmental stages (Dunn et al., 2019) and thus are particularly sensitive to impact from the social environment. Furthermore, most studies to date have been limited to the investigation of the epigenome at a single timepoint. The relationship of the early life social environment to dynamic epigenetic marks over time has only been studied in a few cohorts, such as the Avon Longitudinal Study of Parents and Children cohort (Alfano et al., 2019; Dunn et al., 2019; Liu et al., 2023; Lussier, Zhu, Smith, Cerutti, et al., 2023; Lussier, Zhu, Smith, Simpkin, et al., 2023), Environmental Influence on Ageing cohort (Alfano et al., 2019), the Edinburgh Reproductive Tissue BioBank (Piyasena et al., 2016), and the Northern Manhattan Mothers and Newborns Study of the Columbia Center for Children's Environmental Health cohort (Herbstman et al., 2013). Methods used in these cohorts varied from nonspecific global DNA methylation analyses (Herbstman et al., 2013) to epigenome‐wide arrays, used to investigate hundreds of thousands of individual DNA methylation changes (Alfano et al., 2019; Dunn et al., 2019; Liu et al., 2023; Lussier, Zhu, Smith, Cerutti, et al., 2023; Lussier, Zhu, Smith, Simpkin, et al., 2023). Collective results varied, demonstrating that the timing and duration of childhood experiences of adversity, as well as the timing of epigenetic measures, all affect the relationship. Additionally, promising interventions have been developed to help mitigate the intergenerational impacts of childhood adversity in adults and their children, but the biological changes that may underlie positive outcomes resulting from such interventions are largely unknown.

The present manuscript aims to address the current knowledge gap on treatment effects in mitigating the impact of social adversity on epigenetic changes in young children. Utilizing a sample of mother–child pairs selected for the greater risk of social and relational adversity, we followed two main aims. First, we investigated how potentially stressful/maladaptive environmental experiences in infants and toddlers are related to salivary DNA methylation in three key brain genes and potential biomarkers of psychological risk, brain‐derived neurotrophic factor (*BDNF)*, solute carrier family 6 member 4 (*SLC6A4)*, and nuclear receptor subfamily 3 group C member 1 (*NR3C1*), as well as the transposable genetic element, long interspersed nuclear element‐1 (LINE1). We used a hypothesis‐driven candidate gene approach to maximize statistical power in a smaller cohort. We selected these regions as they have all been identified as central contributors to brain health in both adults and children. DNA methylation at these genes and LINE1 has been previously identified as sensitive to environmental perturbations, including to psychosocial stressors (Oberlander et al., 2008; Stenz et al., 2015; Virani et al., 2012; Wankerl et al., 2014). Second, we sought to explore whether parent–infant intergenerational psychotherapy could interact with these social and relational adversities to alter DNA methylation over time. We hypothesized that adverse social and relational experiences and measures of parenting in the dyads would be associated with DNA methylation levels in the child (and possibly also parent), with direction of association varying by gene, and that psychotherapy effect would interact with adversity to alter DNA methylation levels over time.

## 2 MATERIALS AND METHODS

### 2.1 Participants

**FIGURE 1:**
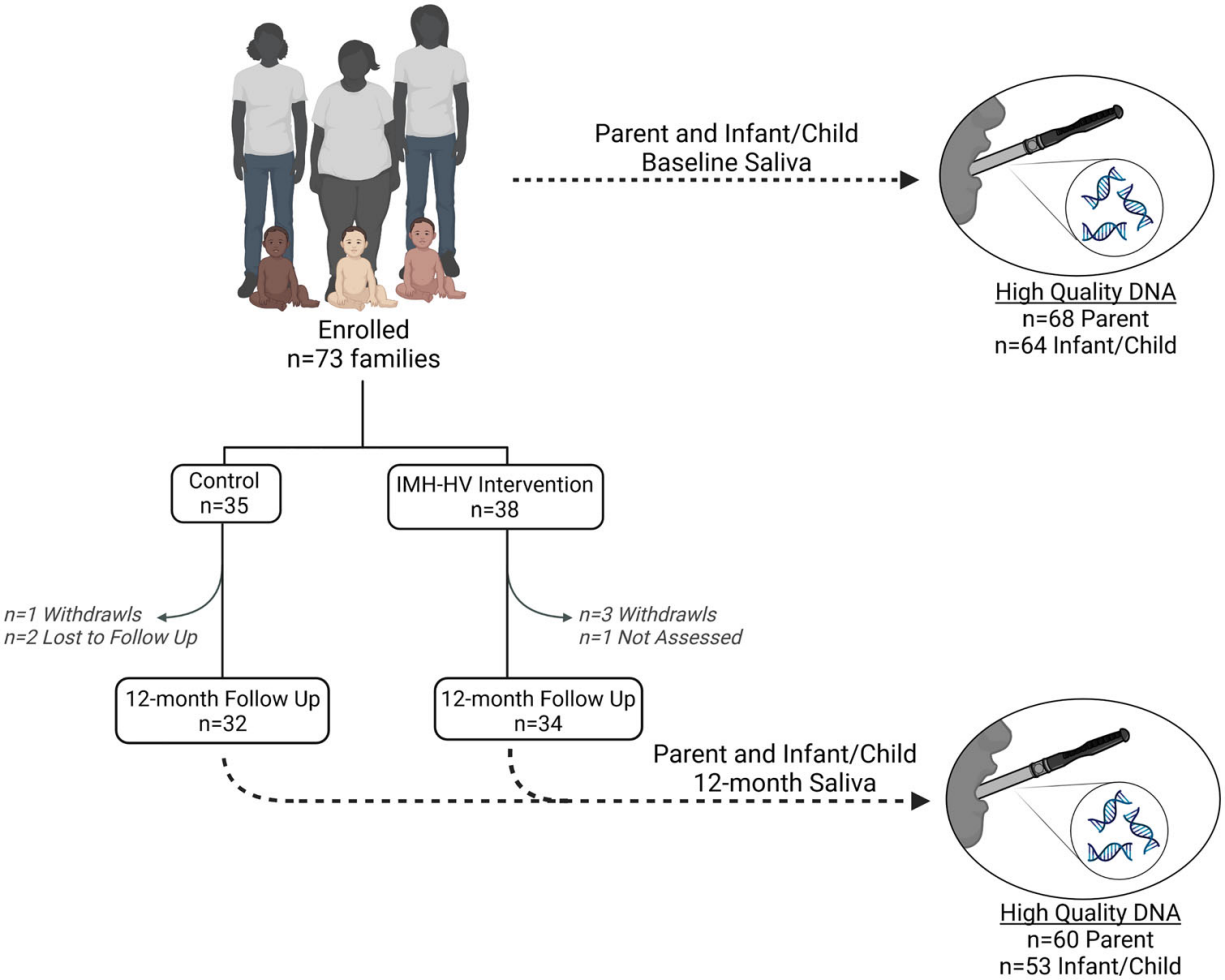
Overall study design. At baseline, 73 families were enrolled and randomized to either control (treatment as usual) or Infant Mental Health Home Visiting (IMH‐HV) intervention. Thirty‐five families were assigned to the control arm, and 38 families were assigned to the intervention arm. At baseline and at the 12‐month follow‐up, saliva samples and measures of parent/child risk were collected. At baseline, we extracted high‐quality saliva DNA adequate for DNA methylation analyses from 68 parents and 64 infants/children. Three infants were not yet born, and one family did not have any high‐quality DNA samples. Between baseline and the 12‐month follow‐up, three families were dropped from the control group, including one withdrawal and two lost to follow‐up. Four families were dropped from the intervention group, including three withdrawals and one who was not assessed. At the 12‐month follow‐up, there were 60 high‐quality parent DNA samples and 53 high‐quality infant/child samples. Table 1 describes passing sample sizes for individual gene assays. *Source*: Created in BioRender.

This study includes data from the Thriving Together: Promoting Early Positive Development study (ClinicalTrials.gov ID NCT03175796, Michigan Medicine Institutional Review Board: HUM00124224). Mothers and their infants/toddlers between 0 and 24 months were recruited at baseline (*n* = 146, 73 pairs, Figure 1) to participate in a randomized control trial evaluating the effectiveness of The Michigan Model of Infant Mental Health Home Visiting (IMH‐HV) parent–infant psychotherapy compared to treatment as usual. Mothers were eligible to participate if they endorsed at least two of the following: (1) a score above cutoff for possible depression on a self‐report screening questionnaire (score >9 on PHQ‐9) (Kroenke et al., 2001), (2) three or more adverse childhood experiences (ACEs) (Felitti et al., 1998), (3) challenges with parenting and/or their child's behavior (parent‐reported), and (4) household eligibility for public assistance (based on parent‐reported family income and household size). Mothers were not eligible for participation if they met screening criteria for active substance use disorders (Brown & Rounds, 1995) or psychosis (Dengenhardt et al., 2005).

### 2.2 Study design

Following the baseline assessment, urn randomization was utilized to assign half of the sample to a treatment‐as‐usual control group (with access to all available community resources) and half to the intervention treatment group. Women assigned to the treatment group received weekly IMH‐HV sessions for up to 12 months with rigorous monitoring of fidelity to the IMH‐HV model (Huth‐Bocks et al., 2020; Weatherston et al., 2020; Weatherston & Tableman, 2015). Sessions were offered as soon as 1 week after the baseline assessment and were scheduled weekly at the preference of the family. Data from baseline and 12 months following study enrollment were used in this manuscript. IMH‐HV sessions were offered for up to 12 months and could have ended just before the final data collection visit or could have ended sooner if the family wished to terminate services sooner (e.g., due to scheduling conflicts or meeting treatment goals). Data collection standardly occurred in the family's home and included verbal administration of self‐report questionnaires, biological sample collection, home environment observation, video‐taped parent–child interaction, and audio‐recorded interviews. Evaluators collecting data were blind to treatment conditions.

### 2.3 Description of intervention

IMH‐HV is a multi‐faceted, multi‐component, needs‐driven parent–infant psychotherapy intervention delivered in the home to parents and is available from pregnancy through age 3 years for the child. IMH‐HV services are delivered by master's prepared clinicians who are required to hold an Infant Mental Health endorsement assuring high quality and fidelity to the standardized IMH‐HV model based on a written curriculum. Like many home visiting programs, IMH‐HV addresses contextual needs, connection to resources, psychoeducation, and developmental guidance. The intervention itself is intended to be delivered with any primary caregivers of young children (e.g., mothers, fathers, extended family members, foster parents, to name a few), and at times more than one parent/caregiver is present during a session. In our research study, we limited participation to mothers only. A unique component of IMH‐HV is infant–parent psychotherapy (IPP), which allows providers to observe patterns of interactions between parent and their infant or toddler and use these observations to promote the parents’ reflections on their parenting beliefs and own upbringing, thus “bridging” own relational experiences with one's own family of origin to the parenting style with one's own child in the here and now. These parental reflections on potentially own conflictual relational experiences when growing up may promote healing for the parent and build the foundation for an undistorted and empathic understanding of one's own child's developmental needs in parenting. IPP guides therapists in assessing for and addressing relational unresolved trauma or loss in the parent that might influence their relationship with their young child. Throughout the study, fidelity to the model and the appropriate use of the multiple therapy components were monitored and maintained.

### 2.4 Metrics

#### 2.4.1 Adversity measures

Baseline adversity measures for mothers were defined by responses to self‐report questionnaires regarding demographics and traumatic life experiences. A demographics questionnaire assessed household socio‐economic, demographic, and maternal and child health information. A cumulative index score for characteristics related to structurally driven inequities (SDIs) was created and included variables: (1) having a household income less than 200% of the federal poverty threshold, (2) receiving less than an education level of a high school degree or its equivalent (e.g., GED), (3) being currently unmarried, (4) having four or more children under age 6 living in the home, and (5) identifying with non‐White racial background (Black or African American, American Indian or Alaskan Native, and/or Latino Hispanic ethnicity). Scores on this index are a sum of the total number endorsed, ranging from 0 to 5.

The ACE Questionnaire is a 10‐item survey that assesses whether specific interpersonal traumatic events were experienced before the age of 18 (Felitti et al., 1998). Items assess for emotional and physical neglect, emotional, physical, and sexual abuse, and various indicators of household dysfunction, including having an incarcerated parent or living with someone with a substance use disorder.

The Life Events Checklist‐5 (Weathers et al., 2013) is a 17‐item measure to assess experience with potentially traumatic events throughout the lifespan, both as a child and adult. For each event, respondents indicated experience and exposure to such events, such as natural disasters, physical or sexual assault, and life‐threatening illnesses or injuries.

Baseline adversity and home social environment measures for infants and toddlers were defined by maternal responses to self‐report questionnaires and observer ratings. Parenting attitudes and beliefs were assessed with the Brief Child Abuse Potential (BCAP) Inventory (Ondersma et al., 2005) and the Adult‐Adolescent Parenting Inventory (AAPI) (Bavolek & Keene, 1999). The BCAP is a 34‐item questionnaire that consists of agree/disagree statements to assess a mother's likelihood for abuse or neglect to their child. Both the total score and cut‐off of 12 indicating elevated risk for engaging in child abuse were utilized (Ondersma et al., 2005). Internal consistency was strong (*α* = 0.85). The AAPI is a 40‐item questionnaire used to assess parenting attitudes. Subscales include maternal expectations of children, lack of empathy, belief in value of physical punishment, parent–child role reversal, and oppression of children's power and independence. Internal consistency on the total measure was good (*α* = 0.80), and subscale reliability was acceptable (*α* ranged from 0.74 to 0.87). Higher values on the BCAP scales and lower values on the AAPI scales represent parenting beliefs and attitudes associated with more adverse child outcomes.

#### 2.4.2 Home quality and parenting measures

In addition to questionnaires, study evaluators completed post‐visit ratings based on observations made during their time spent with the family in their homes during data collection assessments. Stimulating and supportive quality of the home environment for the child was assessed by the Infant‐Toddler Home Observation for Measurement of the Environment (HOME) (Caldwell & Bradley, 2003). Total score and scale scores for two subscales of the HOME were utilized: acceptance of the child and parental responsivity.

Parenting sensitivity was assessed using a 25‐item shortened version of the maternal behavior Q‐set (MBQS) (Tarabulsy et al., 2009). Evaluators were trained and demonstrated reliability (ICC ≥ 0.80) on the shortened MBQS, which involves sorting 25 items describing observed maternal behavior into five piles. This sort results in a score that indicates the level of positive correlation with a profile score of a sensitive, responsive parent.

#### 2.4.3 Biological measures

##### 2.4.3.1 Sample collection

Saliva samples were collected from both mothers and infants/toddlers at the baseline and 12‐month follow‐up using Oragene Kits (DISCOVER, DNA Genotek; OGR‐500 for adults, and OGR‐575 for infants and toddlers). Mothers spit into the Oragene funnel until saliva reached the fill line. For infants and toddlers, evaluators swabbed the inside cheek and gums for 30 s prior to wringing out saliva into the tube. This was repeated with the same sponge until the fill line was reached. Approximately 2 mL for mothers and 0.75 mL for infants and toddlers was collected. After sufficient saliva was collected, the sample was then shaken to mix with the preservative, allowing shelf‐stability at room temperature for up to 5 years.

##### 2.4.3.2 DNA extraction and bisulfite conversion

DNA was isolated using a Qiagen Flexigene Kit (Qiagen) and concentrated using a DNA Clean & Concentrator kit (Zymo). Extracted DNA quality and quantity were assayed using a Nanodrop Spectrophotometer (Thermo Fisher Scientific). Extracted DNA was stored at −80°C until bisulfite conversion. Samples with >250 ng of DNA were bisulfite converted using the EpiTect 96‐well Bisulfite Kit (Qiagen), with families randomly assigned to plates. Of the 73 enrolled families, 71 had samples available. One participating family declined providing saliva samples, and one participating family withdrew their consent for use of saliva samples partway through the study. At baseline, 68 mothers and 64 infants and toddlers had sufficient high‐quality DNA for analysis. At 12‐month post‐enrollment, 60 mothers and 53 infants and toddlers had DNA sufficient for analysis. Converted DNA was stored at −20°C until pyrosequencing.

##### 2.4.3.3 Gene selection

Three genes previously linked with psychiatric health and disease and sensitive to perturbation by psychosocial stressors were optimized (Supplement Material S1). *BDNF* (Stenz et al., 2015) and the glucocorticoid receptor, also known as *NR3C1* (Oberlander et al., 2008), genes were identified, and key regions that have been linked to psychosocial stressors in numerous epidemiological studies were selected for investigation. The serotonin transporter, also known as *SLC6A4* (Wankerl et al., 2014), was also selected due to its role in stress response. The family of retrotransposons called LINE1 (Virani et al., 2012) were also assayed. LINE1 is an abundant and mobile retrotransposon that accounts for up to 17% of all human DNA (Lander et al., 2001). To maintain a stable genome, LINE1 should be heavily methylated, and it is often screened as a surrogate for global DNA methylation (Yang et al., 2004).

##### 2.4.3.4 PCR amplification and pyrosequencing

For each gene or genetic element, converted DNA was amplified via polymerase chain reaction (PCR; Supplement Material S2). After PCR, fragments were analyzed using the QIAxcel Advanced System (Qiagen). Methylation of individual cytosine (CpG) sites of interest was measured using standard protocol on a 96‐well PyroMark MD Pyrosequencer (Qiagen), with multiple consecutive CpG sites included for each gene/element. Each 96‐well batch included replicates of 10% of the samples, both 0% and 100% methylated bisulfite‐converted human DNA (Qiagen), and at least one no‐DNA template control. The plate was repeated if duplicated samples were poorly correlated with their matches or any of the controls failed. Pyrograms were examined, and any well with more than one failed CpG for *BDNF* and LINE1 or two failed CpGs for *SLC6A4* and *NR3C1* were removed from analysis. The final number of samples passing quality control for each gene were as follows for mothers: *BDNF *= 55; *SLC6A4 *= 56, *NR3C1 *= 63, LINE1 = 66; children: *BDNF *= 46, *SLC6A4 *= 51, *NR3C1 *= 54, LINE1 = 54.

### 2.5 Statistical analyses

All analyses were conducted in R (version > 4.2). Demographics for mothers and infants and toddlers were computed and compared between control and intervention participants, using the tableone R package, which assesses groupwise differences using *t*‐tests for continuous variables and *χ* ^2^ tests for categorical variables. For DNA methylation levels at each CpG, outliers were identified and removed from the dataset. Across all data, only two datapoints for *BDNF* were outliers (one at CpG 3 for a child at baseline, and one at CpG 2 for a separate child at baseline, assessed on a separate run). Correlation of intragene methylation was determined across dyad samples. Because all CpGs within the same gene/element were correlated (Supplement Material S3), average methylation across CpGs within a given gene was used for analysis.

For mothers, relationships between baseline DNA methylation and baseline outcome measures of adversity, including measures of ACEs, the Life Events Checklist, and demographic characteristics related to SDIs (SDI index), were considered using a linear model, controlling for smoking status (due to known associations with DNA methylation) (Bollepalli et al., 2019) and batch effects. In a longitudinal analysis, main effects of time (baseline v. 12 month) and treatment group on methylation were evaluated using a linear mixed‐effects model with the R package nlme, with a fixed effect for smoking status and batch and a random effect for participant ID (Yuan et al., 2021). Linear mixed‐effects models were similarly used to evaluate relationships between methylation and baseline experiences of adversity, including ACEs, Life Events, and SDI index measures. Interactions between *treatment* × *time* were evaluated using the same mixed‐effects model.

For infants and toddlers, relationships between baseline methylation and baseline outcome measures, including child abuse potential, measures of parenting sensitivity, and home environment, were considered using a linear model, controlling for child sex (Solomon et al., 2022), child age (Wikenius et al., 2019), maternal smoking status (Joubert et al., 2016), all due to known relationship with DNA methylation, and batch. Main effects of time and treatment and interactions between *treatment* × *time* were then evaluated using a linear mixed‐effects model with the same predictor and outcome variables as above, controlling for the fixed effects of child sex, child age, maternal smoking status, and batch, with a random effect for participant ID. Main effects of baseline adversity and home social environment measures and repeated methylation over time were also considered. Finally, for adversity and social measures that were related to methylation, interactions between *treatment* × *adversity/social environment* were assessed using a repeated‐measures, linear mixed effects model with the same predictor, outcome, and controlled variables as above. Reduced models, controlling for only child sex, age, and batch were assessed for all models. Because we chose the candidate genes with strong justification, we did not control for multiple comparisons (Rothman, 1990).

## 3 RESULTS

### 3.1 Demographics

Demographics are reported in Table 1. There were no differences in baseline demographics, psychological measures, or DNA methylation among treatment groups (*p* > .05).

**TABLE 1:** Baseline demographic and psychological data.

	Total cohort^*^	Treatment as usual controls	Intervention treatment
***N***	**73**	**35**	**38**
**Parents**			
Age (years)	32.01 (5.66)	32.80 (5.19)	31.25 (6.07)
Smoking (% smokers)	9.7% (7)	6.1% (2)	13.2% (5)
*Race*			
White	70.8% (51)	66.7% (22)	73.1% (28)
Black or African American	33.3% (24)	33.3% (11)	34.3% (13)
Asian	1.4% (1)	3% (1)	0% (0)
American Indian or Alaska Native	2.8% (2)	0% (0)	5.3% (2)
*Ethnicity*			
Hispanic or Latino	6.9% (5)	3% (1)	10.5% (4)
Arab or Arab American	2.8% (2)	3% (1)	2.6% (1)
ACEs (sum)	3.61 (2.40)	3.67 (2.50)	3.66 (2.30)
Life event happened (sum)	2.65 (2.09)	2.70 (2.08)	2.68 (2.11)
Life event witnessed (sum)	1.76 (1.86)	1.67 (1.85)	1.89 (1.89)
SDI index score	1.46 (1.30)	1.45 (1.33)	1.45 (1.31)
*BDNF* (average methylation)	4.28 (1.98)	4.24 (1.67)	4.32 (2.22)
*SLC6A4* (average methylation)	4.80 (2.48)	5.03 (2.33)	4.58 (2.63)
*NR3C1* (average methylation)	4.72 (3.11)	5.17 (3.20)	4.34 (3.02)
LINE1 (average methylation)	75.78 (2.96)	75.68 (3.35)	75.88 (2.61)
**Children**			
Age at 12‐mo post‐enroll. (months)	23.66 (7.32)	25.18 (7.27)	22.42 (7.29)
Sex (% female)	56.9% (41)	63.6% (21)	50.0 (19)
BCAP—total (sum)	9.10 (5.15)	8.94 (4.69)	9.37 (5.57)
BCAP—12+ (% above 12)	31.9% (23)	30.3% (10)	34.2% (13)
AAPI—expectations (score)	23.11 (4.55)	22.97 (4.50)	23.32 (4.69)
AAPI—empathy (score)	41.18 (4.95)	40.82 (5.15)	41.79 (4.49)
AAPI—punishment (score)	43.08 (7.87)	43.33 (7.45)	43.00 (8.36)
AAPI—role (score)	29.04 (4.50)	29.15 (4.37)	29.13 (4.59)
AAPI—power (score)	21.97 (2.26)	22.06 (2.32)	21.97 (2.22)
HOME—total (score)	15.12 (1.75)	15.19 (1.75)	15.21 (1.56)
HOME—acceptance (score)	5.73 (1.20)	5.66 (1.26)	5.82 (1.16)
HOME—responsivity (score)	9.39 (1.07)	9.53 (0.76)	9.39 (1.06)
MBQS (score)	0.51 (0.42)	0.49 (0.42)	0.56 (0.39)
*BDNF* (average methylation)	4.28 (2.75)	4.40 (3.04)	4.17 (2.51)
*SLC6A4* (average methylation)	4.93 (3.68)	5.13 (3.38)	4.70 (4.07)
*NR3C1* (average methylation)	4.36 (3.32)	4.23 (2.87)	4.48 (3.74)
LINE1(average methylation)	68.10 (4.35)	68.35 (5.00)	67.73 (3.61)

*Note*: Reported in either percent (count) or mean (SD). Continuous variables were assessed with *t* tests, and categorical variables were assessed with *χ* ^2^ tests. All results were not significantly different between groups (*p* < .05). Passing sample sizes for DNA methylation in parents are as follows: *BDNF *= 55; *SLC6A4 *= 56; *NR3C1 *= 63; LINE1 = 66. Passing sample sizes for DNA methylation in children are as follows: *BDNF *= 46; *SLC6A4 *= 51; *NR3C1 *= 54; LINE1 = 54.

Abbreviations: AAPI, Adult–Adolescent Parenting Inventory; ACE, Adverse Childhood Experiences; BCAP, Brief Child Abuse Potential; BDNF, brain‐derived neurotrophic factor; HOME, Home Observation for Measuring the Environment; LINE1, long interspersed nuclear element‐1; MBQS, maternal behavior Q‐Set; NR3C1, nuclear receptor subfamily 3 group C member 1; SDI, structurally driven inequities; SLC6A4, solute carrier family 6 member 4.

^*^One family dropped from the overall cohort due to poor DNA samples.

In Aim 1, we assessed the baseline association between stressful/maladaptive environmental experiences and salivary DNA methylation in *BDNF*, *SLC6A4*, and *NR3C1*, and LINE1, among our mother–infant dyads. Moving to the second aim, we investigated the longitudinal pattern of associations between adversities and DNA methylation over time and the effect of psychotherapy exposure in interaction with adversities on DNA methylation. Below, we present the results of Aims 1 and 2 separately for mothers and infants/toddlers.

### 3.2 Mothers

For parents, baseline methylation of *BDNF*, *SLC6A4*, *NR3C1*, or LINE1 was largely not associated with baseline measures of childhood adversity, adverse life experiences, or demographic characteristics related to SDIs (Supplement Material S4). Only LINE1 methylation was marginally associated with having experienced more potentially traumatic events (LEC—happened, *β*: 0.34, *p*:.06, 95% CI: −0.02, 070). When examining repeated measures of mothers’ methylation over time and by treatment group, there were no relationships between baseline adversity and repeated methylation (Supplement Material S4) and no significant interactions between time and treatment (Supplement Material S5).

### 3.3 Infants and toddlers

**FIGURE 2:**
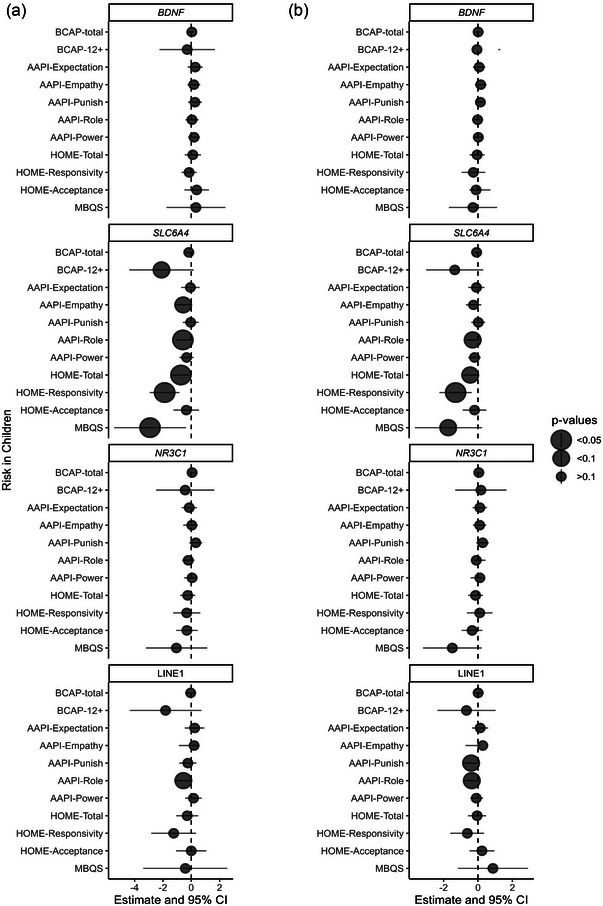
Forest plot of DNA methylation and measures of adversity and the home social environment in children. Part (a) shows baseline methylation model results, and part (b) shows repeated measures of methylation model results. Each panel shows correlation coefficients of average gene methylation at baseline and child measures at baseline. Points are the coefficient estimates, and point size indicates *p* value (larger points are smaller *p* values). Horizontal line indicates 95% confidence interval for the point estimate. Vertical dashed line represented zero. AAPI, Adult–Adolescent Parenting Inventory; BCAP, Brief Child Abuse Potential; HOME, Home Observation for Measuring the Environment; MBQS, maternal behavior Q‐Set.

For infants and toddlers, baseline measures were not related to *BDNF* methylation, but there were some relationships between parenting and the home social environment and methylation of *SLC6A4, NR3C1*, and LINE1 (Figure 2a, Table 2, Supplement Material S6). Specifically, higher scores on a mother‐report measure of child abuse potential were inversely associated with *SLC6A4* methylation (BCAP‐12+ *β*: −2.10, *p*:.07, 95% CI: −4.37, 0.17). Higher (less worrisome) scores on the AAPI and more stimulating and sensitive home environments were inversely associated with *SLC6A4* methylation and LINE1 methylation. Specifically, *SLC6A4* methylation was lower with more maternal empathy (AAPI‐lack of empathy, *β*: −0.57, *p*:.06, 95% CI: −1.16, 0.03), less parent–child role reversal (AAPI—role, *β*: −0.58, *p*:.02, 95% CI: −1.07, −0.10), a more responsive home environment (HOME—total, *β*: −0.72, *p*:.02, 95% CI: −1.33, −0.12; HOME—responsivity, *β*: −1.88, *p*:.001, 95% CI: −2.93, −0.84), and more sensitive parenting (MBQS, *β*: −2.91, *p*:.03, 95% CI: −5.44, −0.37). LINE1 was inversely associated with less mother–child role reversal (AAPI—role, *β*: −0.60, *p*:.05, 95% CI: −1.20, 0.01).

**TABLE 2:** Main effects baseline and repeated DNA methylation and measures of adversity or home social environment in children.

	*BDNF*		*SLC6A4*		*NR3C1*		LINE1	
	Baseline	12‐month post	Baseline	12‐month post	Baseline	12‐month post	Baseline	12‐month post
*Parent‐reported parenting beliefs and values*								
Higher child abuse potential (BCAP, total)	0.04 (0.68) −0.25, 0.17	0.01 (0.91) −0.12, 0.14	−0.17 (0.20) −0.42, 0.09	−0.08 (0.36) −0.26, 0.10	0.06 (0.61) −0.16, 0.27	0.05 (0.50) −0.10, 0.20	−0.04 (0.76) −0.30, 0.22	0.01 (0.87) −0.15, 0.18
Higher child abuse potential (BCAP, 12+)	−0.29 (0.77) −2.24, 1.66	−0.06 (0.93) −1.30, 1.19	−2.10 (0.07)^ −4.37, 0.17	−1.35 (0.11) −3.01, 0.30	−0.43 (0.68) −2.47, 1.62	0.17 (0.82) −1.32, 1.65	−1.81 (0.16) −4.34, 0.72	−0.67 (0.43) −2.36, 1.02
More appropriate expectations of child (AAPI—expectations)	0.28 (0.30) −0.25, 0.80	0.07 (0.71) −0.29, 0.42	−0.06 (0.86) −0.71, 0.59	−0.09 (0.71) −0.56, 0.38	−0.14 (0.62) −0.67, 0.40	0.11 (0.61) −0.31, 0.52	0.24 (0.49) −0.45, 0.92	0.13 (0.59) −0.34, 0.59
More parental empathy (AAPI—empathy)	0.26 (0.28) −0.22, 0.73	0.16 (0.35) −0.18, 0.49	−0.57 (0.06)^ −1.16, 0.03	−0.26 (0.25) −0.70, 0.19	0.04 (0.87) −0.56, 0.47	0.10 (0.48) −0.29, 0.48	−0.20 (0.55) −0.87, 0.47	−0.28 (0.21) −0.72, 0.16
Lower parental value of corporal punishment (AAPI—punish)	0.21 (0.32) −0.21, 0.63	0.14 (0.35) −0.15, 0.42	−0.04 (0.90) −0.60, 0.52	0.02 (0.93) −0.39, 0.42	0.32 (0.16) −0.13, 0.77	0.27 (0.14) −0.09, 0.62	−0.24, (0.42) −0.84, 0.36	−0.40 (0.05)* **−0.79, −0.01**
Lower parent–child role reversal (AAPI—role)	0.05 (0.82) −0.40, 0.51	−0.02 (0.88) −0.33, 0.28	−0.59 (0.02)* **−1.09, −0.09**	−0.31 (0.10) −0.68, 0.06	−0.21 (0.33) −0.65, 0.22	−0.10 (0.54) −0.23, 0.44	−0.56 (0.07)^ −1.16, 0.04	−0.36 (0.06)^ −0.73, 0.01
Lower parental value of oppressing child's power/independence (AAPI—power)	0.20 (0.33) −0.21, 0.61	0.02 (0.88) −0.24, 0.28	−0.33 (0.19) −0.83, 0.17	−0.20 (0.26) −0.56, 0.15	0.06 (0.78) −0.51, 0.38	0.11 (0.48) −0.43, 0.21	0.16 (0.59) −0.42, 0.73	−0.10 (0.69) −0.44, 0.29
*Observed parenting and home environments*								
More positive home environment (HOME—total)	0.11 (0.69) −0.46, 0.69	−0.05 (0.82) −0.48, 0.38	−0.73 (0.02)* **−1.33, −0.12**	−0.45 (0.07)^ −0.94, 0.04	−0.25 (0.35) −0.78, 0.28	−0.14 (0.52) −0.57, 0.29	−0.29 (0.46) −1.07, 0.49	−0.05 (0.84) −0.58, 0.47
Higher maternal responsivity (HOME—responsivity)	−0.15 (0.57) −0.68, 0.38	−0.27 (0.44) −0.95, 0.42	−1.88 (0.001)** **−2.93, −0.84**	−1.30 (0.01)* **−2.24, −0.36**	−0.32 (0.50) −1.28, 0.64	0.10 (0.79) −0.64, 0.84	−1.24 (0.12) −2.82, 0.34	−0.62 (0.21) −1.60, 0.36
Higher maternal acceptance (HOME—acceptance)	0.38 (0.38) −0.48, 1.25	−0.11 (0.71) −0.50, 0.72	−0.35 (0.44) −1.25, 0.55	−0.20 (0.56) −0.88, 0.48	−0.31 (0.42) −1.07, 0.45	−0.34 (0.26) −0.95, 0.26	−0.001 (1.00) −1.07, 1.06	0.23 (0.53) −0.49, 0.95
Higher maternal sensitivity (MBQS)	0.33 (0.74) −1.74, 2.40	−0.29 (0.68) −1.69, 1.11	−2.91 (0.03)* **−5.44, −0.37**	−1.73 (0.08)^ −3.67, 0.24	−1.05 (0.34) −3.20, 1.12	−1.49 **(0.08)^** −3.19, 0.21	−0.42 (0.78) −3.39, 2.55	0.87 (0.40) −1.17, 2.91

*Note*: Model results of DNA methylation regressed on adversity and home social environment measures, controlling for maternal smoking, child age, sex, and batch. Estimates are reported with *p* values in parentheses on the first line and 95% confidence intervals on the second line.

^*p* < .10.

*p < .05.

**p < .01.

Abbreviations: AAPI, Adult‐Adolescent Parenting Inventory; BCAP, Brief Child Abuse Potential; BDNF, brain‐derived neurotrophic factor; HOME, Home Observation for Measuring the Environment; LINE1, long interspersed nuclear element‐1; MBQS, maternal behavior Q‐Set; NR3C1, nuclear receptor subfamily 3 group C member 1; SLC6A4, solute carrier family 6 member 4.

Over time, baseline measures of adversity and the home social environment were associated with repeated measures of methylation in *SLC6A4* and LINE1 (see Figure 2b, Table 2). *SLC6A4* methylation was negatively associated with less mother–child role reversal (AAPI—role; *β*: −0.31, *p*:.10, 95% CI: −0.68, 0.06), a more responsive home and maternal environment (HOME—total; *β*: −0.45, *p*:.07, 95% CI: −0.94, 0.04; HOME—responsivity, *β*: −1.30, *p*:.01, 95% CI: −2.24, −0.36), and more maternal sensitivity (MBQS; *β*: −1.73, *p*:.08, 95% CI: −3.67, 0.24). Repeated LINE1 methylation was negatively associated with less value of corporal punishment (AAPI—punish; *β*: −0.40, *p*:.05, 95% CI: −0.79, −0.01) and less mother–child role reversal (AAPI—role; *β*: −0.36, *p*:.06, 95% CI: −0.73, 0.01). Repeated *NR3C1* methylation was associated with more maternal sensitivity, but only in the reduced model (MBQS; *β*: −1.38, *p*:.10, 95% CI: −3.04, 0.29; Supplement Material S6).

Repeated methylation measures were not associated with time or treatment (Supplement Material S7). There was suggestive evidence, however, of an interaction between time and treatment for *BDNF* (*β* for interaction: −2.10, *p*:.07, 95% CI: −4.38, 0.18) (Supplement Material S8).

All measures associated with methylation in baseline or in repeated measures models were then examined with an interaction of treatment. When assessing the interaction of these measures by treatment, there was no evidence of an interaction between measures of parenting or adversity and the intervention term (Table 3).

**TABLE 3:** Repeated DNA methylation and measures of adversity or home social environment in children with treatment interaction.

	Adversity/Home/Social measure	Treatment	Interaction with treatment
***SLC6A4***			
*Parent‐reported parenting beliefs and values*			
Higher child abuse potential (BCAP, 12+)	−0.39 (0.74) −2.70, 1.93	−0.50 (0.60) −2.38, 1.39	−1.73 (0.30) −5.07, 1.61
More parental empathy (AAPI—empathy)	−0.58 (0.84) −0.64, 0.52	0.82 (0.75) −4.25, 5.89	−0.35 (0.44) −1.27, 0.56
Lower parent–child role reversal (AAPI—role)	−0.35 (0.17) −0.86, 0.16	−1.94 (0.48) −7.38, 3.50	0.14 (0.74) −0.66, 0.93
*Observed parenting and home environments*			
More positive home and parental environment (HOME—total)	−0.53 (0.08) −1.12, 0.06	−7.84 (0.36) −24.99, 9.31	0.44 (0.43) −0.67, 1.54
Higher maternal responsivity (HOME—responsivity)	−1.06 (0.05) −2.09, −0.02	7.26 (0.55) −17.10, 31.62	−0.85 (0.50) −3.37, 1.67
Higher maternal sensitivity (MBQS)	−1.67 (0.20) −4.27, 0.93	−1.23 (0.35) −3.83, 1.36	0.18 (0.93) −3.72, 4.08
***NR3C1***			
*Observed parenting and home environments*			
Higher maternal sensitivity (MBQS)	−1.35 (0.28) −3.80, 1.11	−0.43 (0.71) −2.77, 1.90	−0.27 (0.88) −3.73, 3.19
**LINE1**			
*Parent‐reported parenting beliefs and values*			
Lower parental value of corporal punishment (AAPI—punish)	−0.71 (0.02) −1.30, −0.12	−3.13 (0.20) −7.93, 1.67	0.56 (0.15) −0.22, 1.34
Lower parent–child role reversal (AAPI—role)	−0.52 (0.06) −1.07, 0.03	−2.12 (0.45) −7.74, 3.50	0.34 (0.41) −0.48, 0.116

*Note*: Interaction model results of DNA methylation regressed on adversity and home social environment measures × treatment, controlling for maternal smoking, child age, sex, and batch. Estimates and *p* values are reported in parentheses on the first line and the 95% confidence intervals on the second line.

Abbreviations: AAPI, Adult‐Adolescent Parenting Inventory; BCAP, Brief Child Abuse Potential; HOME, Home Observation for Measuring the Environment; LINE1, long interspersed nuclear element‐1; MBQS, maternal behavior Q‐Set; NR3C1, nuclear receptor subfamily 3 group C member 1; SLC6A4, solute carrier family 6 member 4.

## 4 DISCUSSION

Psychological health and wellbeing are dependent upon a wide variety of biological and environmental factors that are intricately interlinked. In early life, the social environment can crosstalk with the epigenome to establish long‐term health and disease risk, but most human studies on this relationship are cross‐sectional or assay a single‐timepoint (for reviews, see Chan et al. (2018), Jiang et al. (2019), and Nothling et al. (2020)). In this study, we assessed the longitudinal epigenetic differences that are (1) associated with adversity and the home social environment in infancy/toddlerhood and parenthood and (2) are differentially altered with intergenerational psychological intervention.

Overall, we did not find any statistically significant associations between adversity and maternal DNA methylation, neither at baseline or over time, nor in relation to treatment effect. This may not be surprising based on the small sample size and the short longitudinal time span in the investigation, and this finding suggests a robustness of epigenetic marks in adults. In contrast, results generated from the infants and toddlers suggest more plasticity in biological marks early in development in response to social environment. Some of these associations did not reach statistical significance and may serve as a possible signal for more complex potential changes in epigenetics during infancy and toddlerhood. In the following paragraphs, we elaborate on our infant/toddler results across the four candidate genes, followed by more general comments contextualizing our findings or lack thereof.

### 4.1 SLC6A4

The serotonin receptor gene, *SLC6A4*, codes for the serotonin transporter protein that transports serotonin out of the synapse, ultimately being responsible for terminating the effect of serotonin in the synapse. DNA methylation of *SLC6A4* is associated with decreased transcription of the serotonin transporter, leading to increased serotonin in the synapse. Changes in serotonin transporter activity have been associated with numerous psychiatric diseases and disorders, including major depressive disorder, autism spectrum disorder, and substance use disorders (Palma‐Gudiel & Fananas, 2017). Methylation of *SLC6A4* has been broadly studied in adults who have been exposed to childhood maltreatment or trauma (Beach et al., 2010; Hossack et al., 2020; Kang et al., 2013; Koenen et al., 2011; Parade et al., 2021; Philibert et al., 2008). In children older than 5 years, studies have found inverse relationships among institutionalization (Non et al., 2016), bullying (Ouellet‐Morin et al., 2013), and early life pain (Chau et al., 2014) and *SLC6A4* methylation, that is, gene hypomethylation and reduced synaptic serotonin in the context of risk. In infants, however, the relationship between early life challenges (preterm birth or pain) and *SLC6A4* methylation status has been mixed (Devlin et al., 2010; Gartstein et al., 2016; Mariani Wigley et al., 2021; Montirosso et al., 2016; Provenzi et al., 2017; Provenzi et al., 2015). Similarly, our infant/toddler results for *SLC6A4* methylation and association with risk were mixed. Like several previously published results, we found decreased methylation of *SLC6A4* associated with infants’/toddlers’ exposure to early life challenges (i.e., parent‐self‐reported higher child abuse potential scores). Somewhat surprisingly, we also found an association between decreased methylation of *SLC6A4* and aspects of responsive parenting or positive home environment in our infant/toddler sample, seemingly contradicting the previous finding. These relationships were robust over time. In review of the literature, this finding may be in line with a previous study, where Provenzi et al. (2017) reported on maternally sensitive parenting moderating the association between infant *SLC6A4* methylation status and risk. Accordingly, although parenting quality may be an important aspect of social environment shaping methylation, it is certainly not exclusive. In this regard, our findings support prior work on *SLC6A4* hypomethylation in the context of social risk, even in the presence of potential moderation via parental sensitivity.

### 4.2 LINE1

LINE1 are repetitive elements that represent approximately 17%–18% of the human genome and have a copy number of approximately half a million. It is well established that the methylation status of these LINE1 repeats serves as a surrogate measure of global DNA methylation and that consequences of LINE1 hypomethylation are genomic instability and alteration of gene expression (Kitkumthorn & Mutirangura, 2011). In the infants’/toddlers’ analyses, we found that higher methylation in LINE1 was associated with responsive parenting attitudes. Prior work linked LINE1 methylation to neurodevelopment and disorders, such as autism spectrum disorder, major depressive disorder, and schizophrenia (Suarez et al., 2018). Another prior study also reported increased methylation could be an indication of restoration in an intervention group of preterm infants receiving higher maternal attention (Fontana et al., 2021). In this regard, our results are in line with previously published work.

### 4.3 NR3C1

The glucocorticoid receptor gene, also known as *NR3C1*, codes for the protein that builds the glucocorticoid receptors, ubiquitous across most brain regions and cell types, and responsible for binding with cortisol and other glucocorticoids. These receptors help regulate the stress response across the life course. Early life adversity has been found to be associated with hypermethylation of *NR3C1* in both animal and human studies (Turecki & Meaney, 2016); specifically, stress exposure in childhood is linked with greater methylation of *NR3C1* in adults and children. For example, Tyrka et al. (2015) have found hypermethylation of *NR3C1* in association with early child maltreatment in preschool‐aged children.

In our study, we did not confirm these prior results. We observed that methylation in *NR3C1* was largely not associated with our measures of interest. In summary, although *NR3C1* is commonly found to be hypermethylated with increasing incidence of adversity (for review, see Parade et al. (2021)), some studies have reported that *NR3C1* methylation is not linked with adversity or maternal sensitivity (Berretta et al., 2021; Cicchetti et al., 2016; Daskalakis & Yehuda, 2014; Hecker et al., 2016), and our results presented here align with these latter findings.

### 4.4 BDNF

Finally, we investigated *BDNF*, the *BDNF*. Epigenetic changes (i.e., increase in methylation level) within the gene‐binding site responsible for *BDNF* gene expression and its protein transcription activity have effects on learning capacity and memory acquisition (Ikegame et al., 2013; Zheleznyakova et al., 2016). Childhood trauma has been linked with *BDNF* methylation (Weder et al., 2014) and, in animal models, *BDNF* methylation is linked to fear memory consolidation (Lubin et al., 2008). *BDNF* methylation has been linked to adult psychopathology from depression, mood disorders, and personality disorders (D'Addario et al., 2012; Fuchikami et al., 2011; Thaler et al., 2014). More recently, research also shows intergenerational passing, such that infant *BDNF* methylation was associated with maternal childhood trauma and fear expression, especially for male newborns (Pilkay et al., 2020). Similarly, maternal experiences of war trauma and chronic stress prenatally were linked to *BDNF* methylation in placental tissue (Kertes et al., 2016). In our study, we found that treatment over time, but not measures of adversity or the home/parenting environment, related to reduction in *BDNF* methylation in infants and toddlers. This promising result may suggest that, especially for infants of mothers with trauma histories, a reduction in methylation would indicate positive a treatment effect. Future studies should aim to explore this relationship to understand if *BDNF* methylation could be a surrogate biomarker for effective parent–child interventions that promote the health of infants and toddlers.

In summary, our manuscript adds to the growing literature showing that throughout childhood, the epigenome is uniquely responsive to the environment (Wong et al., 2010). During the first year of life, the epigenome is dynamic, with methylation largely increasing across various genes (Wikenius et al., 2019). In monozygotic twins, epigenetic differences suggest that the early life epigenome is specific to the individual but highly connected with early life home events (Li et al., 2022). These reported results reflect the important effects that early life experiences can have on the epigenome, with some effects beginning to show around 5–10 years old. Others have also found that the epigenome in infancy, but not early childhood (2.5 years), is related to differences in parenting (Lewis et al., 2020). Our cohort represented a diverse age group across this time period; at baseline, three infants had not yet been born, and others were up to nearly 2 years old. Although we controlled for child age in our models, the relationships observed are likely still reflecting this dynamic epigenetic response across development. Although we confirmed some prior findings in the literature, the differences in child age across our cohort paired with a small sample size may have precluded some findings.

Our cohort also represents a group of mothers and their children that met criteria for possible depression, endorsed experiences of childhood adversity, parenting challenges, and/or income eligibility, which inherently changes the interpretation of our findings. Nearly all other studies on adverse experiences and the epigenome have focused on comparisons between groups with and without experiences of adversity or challenges in the home social environment. For some of our findings (e.g., *SLC6A4* in children), we found relationships that were opposite of those reported in literature. For example, lower *SLC6A4* methylation is often associated with ACEs (and we confirmed this as well), but our results also suggested that lower *SLC6A4* methylation was associated with more responsive parenting. The differences in this relationship could suggest that *SLC6A4* responds variably to different types of adversity (Daskalakis & Yehuda, 2014); methylation has been proposed to be both a response to stress and trauma and an adaption to promote resilience in those living in more potentially traumatic environments (Koenen et al., 2011). It is also noteworthy that other studies in children using the same DNA source for analysis (saliva) also had lower average *SLC6A4* methylation levels than our study (Chau et al., 2014; Ismaylova et al., 2018; Timothy et al., 2019), suggesting that the associations observed may be distinctive to our cohort and not reflective of the broader population.

In line with this hypothesis, we found that DNA methylation in mothers was largely unchanged or unrelated to baseline adversity measures, contrary to the broader consensus from studies with lower risk control groups. We did find one suggestive relationship at baseline; experiencing more potentially traumatic life events was associated with higher LINE1 methylation. This could represent the most variable and extreme measure for adversity we had in our group or could represent a spurious association. Contrary to prior literature cited above, we did not find any associations between maternal methylation patterns and their ACEs, which could have been related to our recruiting criteria of having multiple ACEs at baseline, selecting for a specific population. After comparing the intervention mothers to the control mothers in our study, we also did not see any changes in methylation patterns as a function of intervention. This result was more expected, as early life methylation in contrast to methylation patterns in adults tends to be more sensitive to the environment (Dunn et al., 2019). Even so, there is evidence that exposures in adulthood, including to some types of psychotherapy, may modify the epigenome (Penadés et al., 2020), and it is possible that changes were occurring at genes that we did not assess. Future studies utilizing larger samples should investigate how psychological interventions may be altering methylation across the life span and investigate moderators of effect (e.g., sex or exposure to concurrent adversity).

### 4.5 Limitations and future directions

Overall, this study was one of the largest intervention trials to date that longitudinally tracked child and adult DNA methylation in saliva and in relationship to adversity and the home social environment and to an intervention exposure. Our results somewhat agree with other clinical trials that demonstrate an epigenetic response to psychological interventions in infants (Euclydes et al., 2022; Hoye et al., 2020) and children (Bleker et al., 2019; Brody et al., 2016; Cicchetti et al., 2016) at risk of experiencing adversity, but many of our measures were not associated with DNA methylation. We assayed results in saliva, which is readily accessible, but cannot necessarily be used to infer DNA methylation in the central nervous system or other biologically relevant tissues. It remains essential to understand how methylation patterns related to social environments respond in specific developmental stages (Wikenius et al., 2019), differ according to individuals (Li et al., 2017), and are altered in body tissues and fluids, such as the brain, blood, and saliva (Bakulski et al., 2016).

The findings within this article represent a unique examination of the effects of therapy for mothers and their children who have either already experienced, or are at higher risk for, exposure to adversity, potentially revealing novel insights for future investigation of interventions with populations facing adversity. As such, these results are not generalizable to broader populations but could hold valuable insight into how specialized populations respond to therapy designed to mitigate intergenerational stress and trauma. Going forward, careful studies with the support of interested communities (Goldenberg et al., 2013) are needed to confirm and elucidate the biological significance of the longitudinal epigenetic differences reported here. Future work should attempt to replicate these findings in larger populations, with observation and measurement of epigenetic markers conducted for longer periods of time.

## AUTHOR CONTRIBUTIONS

**Rebekah Petroff**: Writing—original draft; writing—review and editing; visualization. **Jennifer Jester**: Methodology; formal analysis; writing—review and editing. **Jessica Riggs**: Investigation; writing—review and editing. **Emily Alfafara**: Investigation; writing—review and editing. **Katherine Springer**: Investigation; writing—review and editing. **Natalie Kerr**: Investigation; writing—review and editing. **Meriam Issa**: Investigation; writing—review and editing. **Alanah Hall**: Investigation; writing—review and editing. **Katherine Rosenblum**: Conceptualization; methodology; writing—review and editing; supervision. **Jaclyn Goodrich**: Conceptualization; methodology; writing—review and editing; supervision. **Maria Muzik**: Conceptualization; methodology; writing—review and editing; supervision.

## CONFLICT OF INTEREST STATEMENT

The authors declare no conflicts of interest.

### PEER REVIEW

The peer review history for this article is available at https://publons.com/publon/10.1002/brb3.70035.

## Supporting information



Supporting Information

## Data Availability

The data that support the findings of this study are available on request from the corresponding author. The data are not publicly available due to privacy or ethical restrictions.
