# Supplementary material for: Longitudinal DNA methylation in parent–infant pairs impacted by intergenerational social adversity: An RCT of the Michigan Model of Infant Mental Health Home Visiting
Source: Brain Behav. 2024 Sep 18;14(9):e70035. doi: 10.1002/brb3.70035 (PMC11410872; doi:10.1002/brb3.70035)
Supplement: Supplementary file 1 — Supporting Information [file BRB3-14-e70035-s001.docx]

**Supplement Material 1: Gene Region Details**

|  | ***BDNF*** | ***SLC6A4*** | ***NR3C1*** | **LINE1** |
| --- | --- | --- | --- | --- |
| **Region Name** | Exon IV, promoter | Promoter | Exon 1F, promoter |  |
| **GRCh38 Location** | 11:27701549-27701641 | 17:30236043-30236265 | 5:143403936-143404056 | Many |
| **Length (bp)** | 93 | 223 | 121 | Many |
| **Number of CpGs** | 6 | 7 | 8 | 4 |
| **Adapted From** | Stenz, Zewdie et al. (2015) | Wankerl, Miller et al. (2014) | Oberlander, Weinberg et al. (2008) | Virani, Dolinoy et al. (2012) |

Supplemental References

Oberlander, T. F., J. Weinberg, M. Papsdorf, R. Grunau, S. Misri and A. M. Devlin (2008). "Prenatal exposure to maternal depression, neonatal methylation of human glucocorticoid receptor gene (NR3C1) and infant cortisol stress responses." Epigenetics **3**(2): 97-106.

Stenz, L., S. Zewdie, T. Laforge-Escarra, J. Prados, R. La Harpe, A. Dayer, A. Paoloni-Giacobino, N. Perroud and J. M. Aubry (2015). "BDNF promoter I methylation correlates between post-mortem human peripheral and brain tissues." Neurosci Res **91**: 1-7.

Virani, S., D. C. Dolinoy, S. Halubai, T. R. Jones, S. E. Domino, L. S. Rozek, M. S. Nahar and V. Padmanabhan (2012). "Delivery type not associated with global methylation at birth." Clin Epigenetics **4**(1): 8.

Wankerl, M., R. Miller, C. Kirschbaum, J. Hennig, T. Stalder and N. Alexander (2014). "Effects of genetic and early environmental risk factors for depression on serotonin transporter expression and methylation profiles." Transl Psychiatry **4**(6): e402.

**Supplement Material 2: PCR and Pyrosequencing Protocol Methods**

For PCR, 15 µL of HotStarTaq Master Mix (Qiagen) was added to a solution of DNA, 0.3 µM forward primer, 0.2 µM reverse primer, and RNAase free water for a total reaction volume of 30 µL. DNA fragments were amplified via polymerase chain reaction (PCR), as described below. 25 µL of PCR product was used for pyrosequencing, following standardized manufacturer’s protocols.

|  | ***BDNF*** | ***SLC6A4*** | ***NR3C1*** | **LINE1** |
| --- | --- | --- | --- | --- |
| **Forward Primer (5’-3’)** | GGTAGAGGTAGGGAGATTTTATGTTA | GGGGAGGTTGTATAAAGGAGTA | GGGAAGGAGGTAGAGAGAAAAGAAATTG | TTTGAGTTAGGTGTGGGATATA |
| **Reverse Primer (5’-3’)** | Biotin-AAACTCCCACCCACTTT | Biotin-AAACCTCTAAACTAAACTCACATC | Biotin-CCCCCAACTCCCCAAAA | Biotin-AAAATCAAAAAATTCCCTTTC |
| **Sequencing Primer (5’-3’)** | GTAGGGAGATTTTATGTTAG | GGTTGTATAAAGGAGTAGG | GTAGAGAGAAAAGAAATTGGAGAA | AGTTAGGTGTGGGATATAGT |
| **PCR DNA Input (µL)** | 2 | 4 | 2 | 4 |
| **Thermocycler Reaction** | Step 1: (95°C/15 min)/1 cycle, Step 2: (95°C/30 s, 54°C/30 s, 72°C/60 s)/50 cycles, Step 3: (72°C/5 min)/1 cycle | Step 1: (95°C/15 min)/1 cycle, Step 2: (95°C/30 s, 57°C/30 s, 72°C/60 s)/50 cycles, Step 3: (72°C/5 min)/1 cycle | Step 1: (95°C/15 min)/1 cycle, Step 2: (95°C/45 s, 53°C/45 s, 72°C/45 s)/50 cycles, Step 3: (72°C/5 min)/1 cycle | Step 1: (95°C/15 min)/1 cycle, Step 2: (95°C/30 s, 58°C/30 s, 72°C/60 s)/45 cycles, Step 3: (72°C/5 min)/1 cycle |

**Supplement Material 3: Intragene CpG Site Correlations**

| ***BDNF*** | ***SLC6A4*** |
| --- | --- |
| **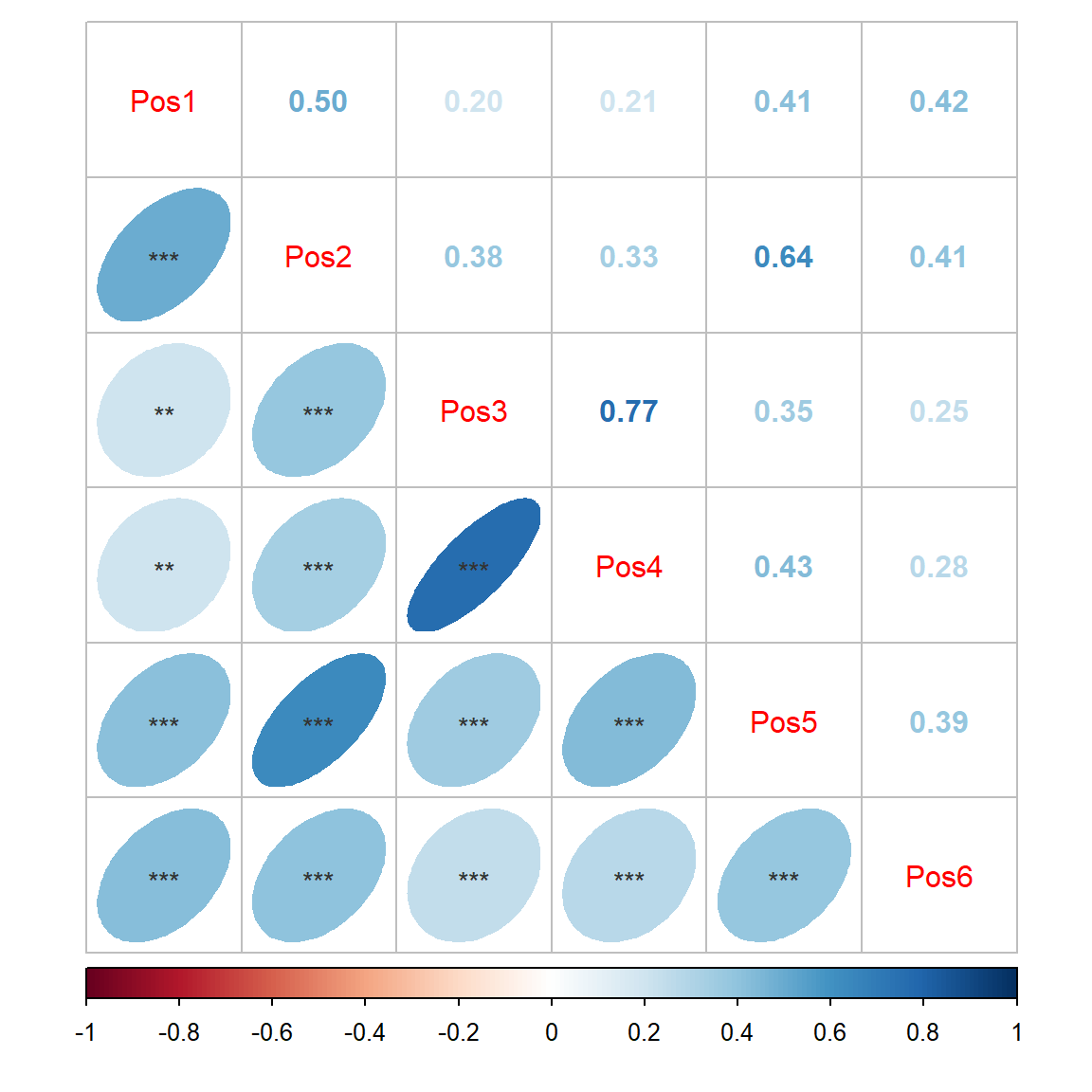** | **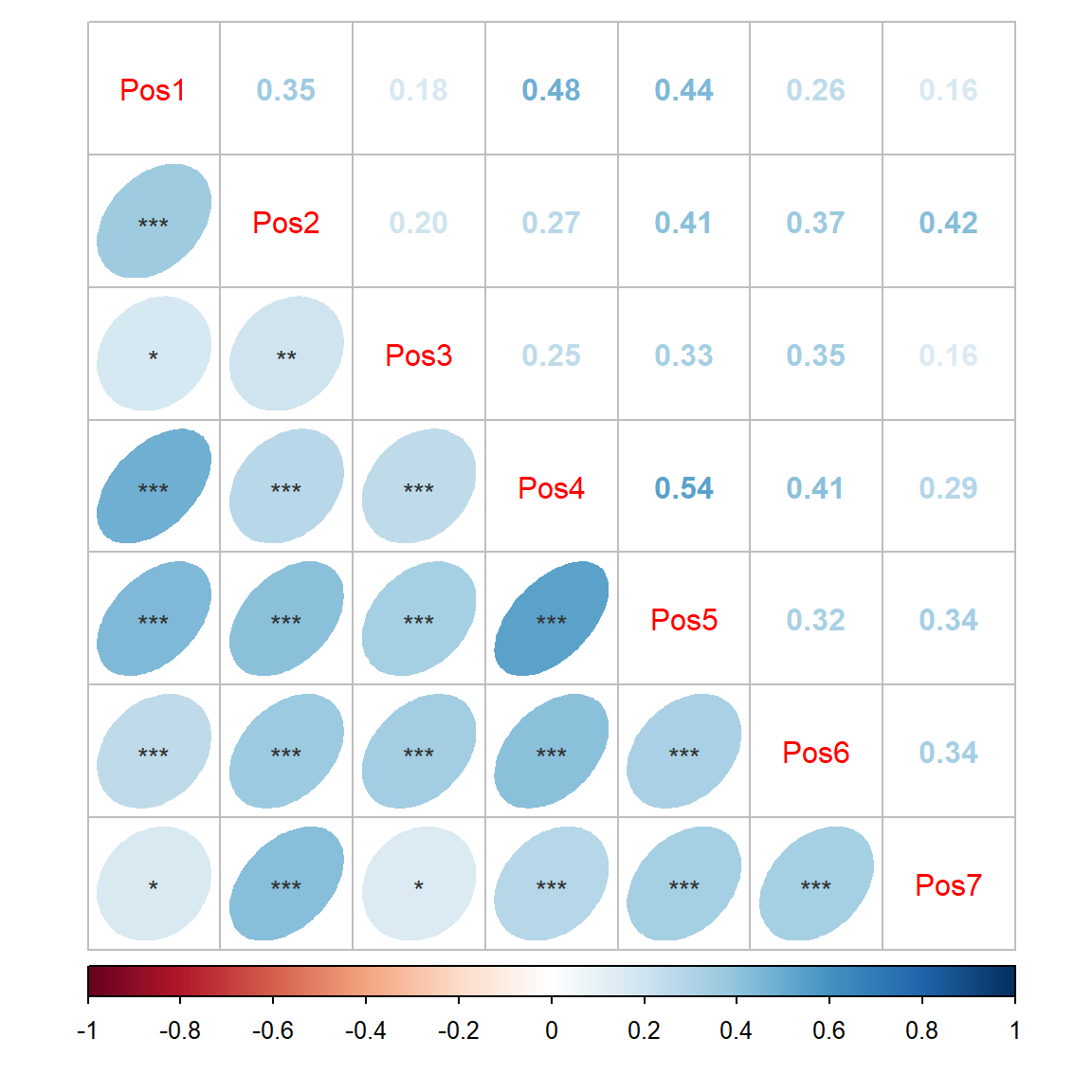** |
| ***NR3C1*** | **LINE1** |
| **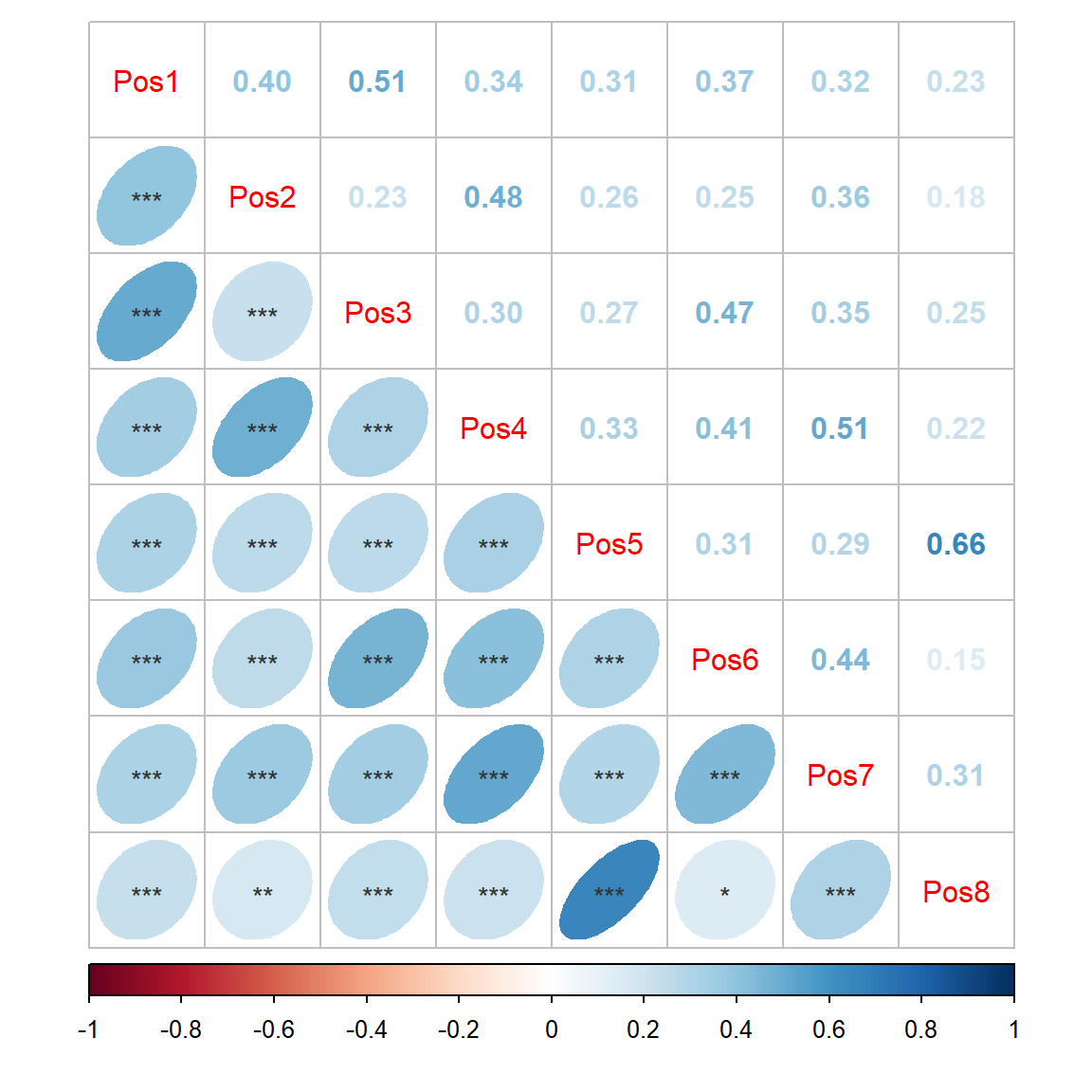** | **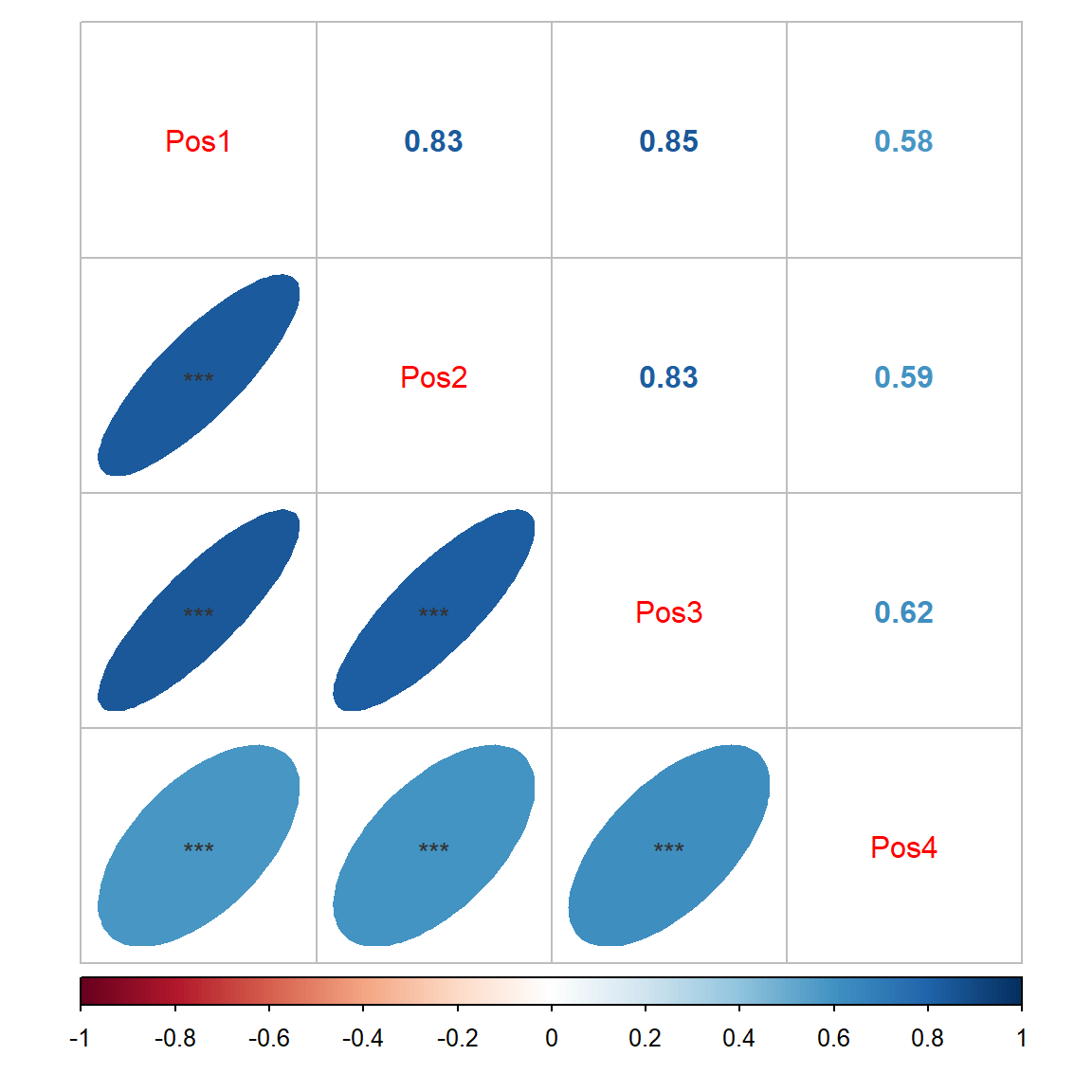** |

Numbers represent the correlation coefficient of two CpGs, which is also indicated by the shade of each oval (blue are positive, red are negative), and the slant of the oval (pointing to the right upper corner is positive; pointing to the right lower corner is negative. Number of stars represent the uncorrected p-value. *p<0.05; **p<0.01; ***p<0.001

**Supplement Material 4:** **Associations between Adversity and DNA Methylation in Parents**

|  | ***BDNF*** | ***SLC6A4*** | ***NR3C1*** | **LINE1** |
| --- | --- | --- | --- | --- |
| Baseline DNA Methylation | | | | |
| Total ACE | -0.13 (0.27) -0.36, 0.10 | 0.09 (0.57)  -0.21, 0.38 | 0.29 (0.48)  -0.05, 0.62 | 0.21 (0.20)  -0.11, 0.53 |
| SDI Index | -0.16 (0.43)  -0.25, 0.58 | 0.16 (0.54)  -0.36, 0.67 | 0.36 (0.25)  -0.25, 0.97 | 0.21 (0.47)  -0.37, 0.79 |
| LEC Happened | 0.11 (0.43)  -0.16, 0.38 | 0.24 (0.21)  -0.14, 0.62 | 0.14 (0.49)  -0.25, 0.53 | *0.34 (0.06)^*  *-0.02, 0.70* |
| LEC Witnessed | -0.01 (0.92)  -0.29, 0.26 | -0.09 (0.67)  -0.50, 0.33 | -0.16 (0.45)  -0.58, 0.26 | -0.03 (0.89)  -0.43, 0.37 |

| **Repeated Measures DNA Methylation** | | | | |
| --- | --- | --- | --- | --- |
| Total ACE | -0.11 (0.25)  -0.30, 0.08 | -0.07 (0.57)  -0.17, 0.31 | 0.13 (0.28)  -0.14, 0.38 | 0.20 (0.17)  -0.09, 0.48 |
| SDI Index | -0.03 (0.74)  -0.27, 0.43 | 0.16 (0.45)  -0.27, 0.58 | 0.24 (0.33)  -0.25, 0.73 | 0.30 (0.25)  -0.21, 0.81 |
| LEC Happened | -0.01 (0.89)  -0.17, 0.29 | 0.09 (0.54)  -0.21, 0.40 | 0.03 (0.87)  -0.29, 0.34 | 0.05 (0.72)  -0.14, 0.52 |
| LEC Witnessed | 0.05 (0.69)  -0.20, 0.30 | -0.08 (0.65)  -0.26, 0.41 | -0.09 (0.60)  -0.45, 0.26 | 0.03 (0.82)  -0.22, 0.50 |

Model results of methylation regressed on adversity measures, controlling for smoking status and batch. Estimates are reported with p-values in parentheses and 95% confidence intervals on the second line. ^denotes p<0.10

**Supplement Material 5: Main Effects and Interaction for Repeated DNA Methylation in Parents: Time and Treatment**

|  | ***BDNF*** | ***SLC6A4*** | ***NR3C1*** | **LINE1** |
| --- | --- | --- | --- | --- |
| Main Effects Model | | | | |
| Time Only | 0.29 (0.43)  -0.46, 1.04 | -0.37 (0.49)  -1.42, 0.68 | 0.79 (0.20)  -0.42, 2.00 | -0.26 (0.66)  -1.45, 0.92 |
| Treatment Only | 0.5 (0.76)  -0.83, 1.14 | -0.28 (0.65)  -1.50, 0.94 | -0.73 (0.25)  -2.00, 0.54 | -0.40 (0.56)  -1.77, 0.98 |
| Interaction Model | | | | |
| Time | 0.30 (0.59)  -0.84, 1.44 | -0.78 (0.32)  -2.32, 0.77 | 0.55 (0.53)  -1.22, 2.32 | 0.27 (0.75)  -1.44, 1.98 |
| Treatment | 0.17 (0.78)  -1.07, 1.42 | -0.62 (0.46)  -2.17, 0.94 | -0.92 (0.30)  -2.64, 0.82 | 0.09 (0.92)  -1.44, 1.98 |
| Time x Treatment | 0.02 (0.98)  -1.54, 1.51 | 0.78 (0.46)  -1.34, 2.90 | 0.42 (0.73)  -2.03, 2.87 | -1.03 (0.39)  -3.39, 1.34 |

Model results of repeated DNA methylation regressed on time and treatment, controlling for maternal smoking and batch. Time and treatment were first independently assessed for main effects and an interaction model was assessed for interaction between time and treatment. Estimates are reported with p-values in parentheses and 95% confidence intervals on the second line.

**Supplement Material 6: Sensitivity Analysis Main Effects for Baseline DNA Methylation in Children**

|  | ***BDNF*** | ***SLC6A4*** | ***NR3C1*** | **LINE1** |
| --- | --- | --- | --- | --- |
| Baseline Methylation | | | | |
| BCAP (total) | -0.02 (0.86)  -0.22, 0.18 | -0.17 (0.16)  -0.41, 0.07 | -0.03 (0.76)  -0.17, 0.23 | -0.04 (0.78)  -0.28, 0.21 |
| BCAP (12+) | -0.03 (0.77)  -2.22, 1.66 | *-2.13 (0.06)^*  *-4.37, 0.12* | -0.47 (0.64)  -2.48, 1.54 | -1.78 (0.16)  -4.28, 0.71 |
| AAPI (Exp) | 0.22 (0.38)  -0.29, 0.74 | -0.04 (0.90)  -0.68, 0.60 | -0.12 (0.65)  -0.65, 0.41 | 0.23 (0.50)  -0.44, 0.91 |
| AAPI (Empathy) | 0.22 (0.34)  -0.25, 0.69 | *-0.54 (0.07)^*  *-1.12, 0.05* | -0.02 (0.93)  -0.53, 0.48 | -0.20 (0.54)  -0.85, 0.45 |
| AAPI (Punish) | 0.16 (0.44)  -0.25, 0.56 | 0.02 (0.96)  -0.51, 0.54 | 0.32 (0.14)  -0.11, 0.74 | -0.21 (0.44)  -0.76, 0.34 |
| AAPI (Role) | 0.06 (0.91)  -0.43, 0.43 | **-0.55 (0.03)***  **-1.04, -0.05** | -0.19 (0.38)  -0.61, 0.24 | *-0.54 (0.07)^*  *-1.12, 0.04* |
| AAPI (Power) | 0.18 (0.39)  -0.23, 0.58 | -0.31 (0.22)  -0.80, 0.19 | 0.05 (0.84)  -0.48, 0.39 | 0.15 (0.60)  -0.41, 0.71 |
| HOME (total) | 0.04 (0.88)  -0.50, 0.59 | **-0.68 (0.02)***  **-1.26, 0.10** | -0.23 (0.38)  -0.75, 0.29 | -0.28 (0.45)  -1.03, 0.47 |
| HOME (responsivity) | -0.15 (0.73)  -1.03, 0.73 | **-1.88 (0.001)****  **-2.92, -0.84** | -0.32 (0.50)  -1.27, 0.62 | -1.24 (0.12)  -2.80, 0.32 |
| HOME (acceptance) | 0.22 (0.58)  -0.58, 1.03 | -0.29 (0.51)  -1.17, 0.59 | -0.26 (0.48)  -1.00, 0.47 | -0.01 (0.99)  -1.02, 1.00 |
| MBQS | 0.18 (0.86)  -1.84, 2.20 | **-2.66 (0.04)***  **-5.14, -0.18** | -0.93 (0.38)  -3.04, 1.18 | -0.42 (0.77)  -3.28, 2.50 |
| Repeated Measures Methylation | | | | |
| BCAP (total) | -0.001 (0.99)  -0.12, 0.12 | 0.09 (0.31)  -0.25, 0.08 | 0.04 (0.58)  -0.10, 0.18 | 0.03 (0.70)  -0.13, 0.19 |
| BCAP (12+) | -0.08 (0.90)  -1.31, 1.16 | -1.35 (0.10)  -3.00, 0.27 | 0.15 (0.84)  -1.32, 1.62 | -0.62 (0.46)  -2.30, 1.06 |
| AAPI (Exp) | 0.08 (0.64)  -0.27, 0.43 | -0.09 (0.71)  -0.55, 0.38 | 0.11 (0.59)  -0.30, 0.52 | 0.11 (0.65)  -0.35, 0.56 |
| AAPI (Empathy) | 0.16 (0.31)  -0.16, 0.48 | -0.23 (0.29)  -0.66, 0.20 | 0.10 (0.59)  -0.28, 0.48 | -0.31 (0.16)  -0.73, 0.12 |
| AAPI (Punish) | 0.14 (0.30)  -0.13, 0.41 | - 1. (0.81)   -0.33, 0.42 | 0.25 (0.13)  -0.08, 0.58 | **-0.40 (0.03)***  **-0.76, -0.04** |
| AAPI (Role) | 0.004 (0.98)  -0.28, 0.28 | -0.28 (0.12)  -0.64, 0.08 | 0.11 (0.51)  -0.22, 0.43 | **-0.38 (0.04)***  **-0.74, -0.01** |
| AAPI (Power) | 0.03 (0.81)  -0.22, 0.28 | -0.19 (0.28)  -0.54, 0.16 | -0.10 (0.51)  -0.42, 0.21 | -0.10 (0.60)  -0.46, 0.26 |
| HOME (total) | -0.004 (0.98)  -0.39, 0.39 | *-0.42 (0.08)^*  *-0.90, 0.06* | -0.12 (0.57)  -0.54, 0.30 | -0.10 (0.71)  -0.61, 0.42 |
| HOME (responsivity) | -0.25 (0.47)  -0.93, 0.43 | **-1.24 (0.01)***  **-2.15, -0.33** | 0.10 (0.78)  -0.63, 0.84 | -0.64 (0.20)  -1.61, 0.34 |
| HOME (acceptance) | 0.14 (0.59)  -0.39, 0.68 | -0.18 (0.59)  -0.84, 0.48 | -0.30 (0.31)  -0.89, 0.28 | 0.14 (0.69)  -0.56, 0.84 |
| MBQS | -0.22 (0.74)  -1.59, 1.15 | *-1.63 (0.09)^*  *-3.55, 0.28* | *-1.38 (0.10)^*  *-3.04, 0.29* | 0.67 (0.50)  -1.32, 2.70 |

Model results of DNA methylation regressed on adversity and home social environment measures, controlling for child age, sex, and batch. Estimates are reported with p-values in parentheses and 95% confidence intervals on the second line. ^p<0.10; *p<0.05; **p<0.01

**Supplement Material 7: Main Effects for Repeated DNA Methylation in Children: Time and Treatment**

|  | ***BDNF*** | ***SLC6A4*** | ***NR3C1*** | **LINE1** |
| --- | --- | --- | --- | --- |
| Full Main Effects Models | | | | |
| Time | -0.52 (0.36)  -1.68, 0.64 | -0.13 (0.86)  -1.60, 1.35 | 0.68 (0.28)  -0.58, 1.94 | 0.43 (0.56)  -1.04, 1.90 |
| Treatment | -0.80 (0.18)  -1.98, 0.38 | -1.15 (0.15)  -2.71, 0.41 | -0.63 (0.38)  -2.05, 0.79 | 0.11 (0.90)  -1.57, 1.78 |
| Reduced Main Effects Models | | | | |
| Time | -0.53 (0.35)  -1.68, 0.62 | -0.12 (0.87)  -1.60, 1.35 | 0.68 (0.28)  -0.57, 1.94 | 0.46 (0.53)  -1.01, 1.93 |
| Treatment | -0.82 (0.17)  -1.99, 0.35 | -1.17 (0.13)  -2.71, 0.36 | -0.65 (0.36)  -2.05, 0.75 | 0.17 (0.84)  -1.49, 1.83 |

Model results of repeated DNA methylation regressed on time and treatment, controlling for maternal smoking, child sex, age, and batch in the full models, and child sex, age, and batch in the reduced models. Time and treatment were independently assessed for main effects. Estimates are reported with p-values in parentheses and 95% confidence intervals on the second line.

**Supplement Material 8: Interaction Effects for Repeated Methylation in Children: Time and Treatment**

|  | ***BDNF*** | ***SLC6A4*** | ***NR3C1*** | **LINE1** |
| --- | --- | --- | --- | --- |
| Full Interaction Model | | | | |
| Time | 0.55 (0.49)  -1.07, 2.18 | 0.31 (0.76)  -1.71, 2.32 | 1.30 (0.15)  -0.50, 3.10 | -0.41 (0.21)  -2.40, 1.58 |
| Treatment | 0.16 (0.84)  -1.47, 1.71 | -0.71 (0.50)  -2.79, 1.37 | -0.10 (0.92)  -1.93, 1.74 | -0.79 (0.47)  -2.98, 1.40 |
| Time x Treatment | *-2.10 (0.07)^*  *-4.38, 0.18* | -0.94 (0.53)  -3.96, 2.09 | -1.17 (0.34)  -3.63, 1.30 | 1.86 (0.21)  -1.07, 4.80 |
| Reduced Interaction Model | | | | |
| Time | 0.55 (0.49)  -1.07, 2.17 | 0.30 (0.76)  -1.72, 2.32 | 1.30 (0.15)  -0.50, 3.10 | -0.41 (0.68)  -2.39, 1.58 |
| Treatment | 0.15 (0.85)  -1.39, 1.68 | -0.75 (0.47)  -2.81, 1.30 | -0.10 (0.91)  -1.93, 1.72 | -0.75 (0.49)  -2.93, 1.43 |
| Time x Treatment | *-2.11 (0.07)^*  *-4.40, 0.16* | -0.92 (0.54)  -3.95, 2.12 | -1.18 (0.34)  -3.64, 1.29 | 1.91 (0.20)  -1.03, 4.84 |

Model results of repeated DNA methylation regressed on time and treatment, controlling for child sex, age, batch, and maternal smoking in the full models, and batch, child sex, and age in the reduced models. Time and treatment were assessed for with an interaction term. Estimates are reported with p-values in parentheses and 95% confidence intervals on the second line of each cell. ^p<0.10
